# Caracterización clínica y funcional de pacientes con atrofia muscular espinal en el centro-occidente colombiano

**DOI:** 10.7705/biomedica.6178

**Published:** 2022-05-01

**Authors:** Natalia Cardona, Sandra Jhoana Ocampo, Jorge Mario Estrada, María Isabel Mojica, Gloria Liliana Porras

**Affiliations:** 1 Centro de Enfermedades Huérfanas - ComSentido, Salud Comfamiliar, Comfamiliar Risaralda, Pereira, Colombia Centro de Enfermedades Huérfanas - ComSentido, Salud Comfamiliar Comfamiliar Risaralda Pereira Colombia; 2 Grupo de Investigación Salud Comfamiliar, Comfamiliar Risaralda, Pereira, Colombia Comfamiliar Risaralda Pereira Colombia; 3 Maestría en Genética Humana, Universidad Nacional de Colombia, Bogotá, D.C., Colombia Universidad Nacional de Colombia Universidad Nacional de Colombia Bogotá, D.C. Colombia

**Keywords:** atrofia muscular espinal, enfermedades raras, fisioterapia, Muscular atrophy, spinal, rare diseases, physical therapy specialty

## Abstract

**Introducción.:**

La atrofia muscular espinal es una enfermedad neurodegenerativa huérfana de origen genético que afecta las neuronas motoras del asta anterior de la médula espinal, y produce atrofia y debilidad muscular. En Colombia, son pocos los estudios publicados sobre la enfermedad y no hay ninguno con análisis funcional.

**Objetivo.:**

Caracterizar clínica y funcionalmente una serie de casos de atrofia muscular espinal del centro-occidente colombiano.

**Materiales y métodos.:**

Se hizo un estudio descriptivo transversal, entre el 2007 y el 2020, de pacientes con diagnóstico clínico y molecular de atrofia muscular espinal que consultaron en el centro de atención. La evaluación funcional se realizó con las escalas Hammersmith y *Chop Intend*. En la sistematización de los datos, se empleó el programa Epi-Info, versión 7.0.

**Resultados.:**

Se analizaron 14 pacientes: 8 mujeres y 6 hombres. La atrofia muscular espinal más prevalente fue la de tipo II, la cual se presentó en 10 casos. Se encontró variabilidad fenotípica en términos de funcionalidad en algunos pacientes con atrofia muscular espinal de tipo II, cinco de los cuales lograron alcanzar la marcha. La estimación de la supervivencia fue de 28,6 años.

**Conclusiones.:**

Los hallazgos en el grupo de pacientes analizados evidenciaron que los puntajes de la escala de Hammersmith revisada y expandida, concordaron con la gravedad de la enfermedad.

## Abstract (en)

**Introduction:**

Spinal muscular atrophy is a rare genetic neurodegenerative disorder affecting the motor neurons of the anterior horn of the spinal cord, which results in muscle atrophy and weakness. In Colombia, few studies have been published on the pathology and none with functional analysis.

**Objective:**

To characterize clinically and functionally some cases of spinal muscular atrophy patients from Central-Western Colombia.

**Materials and methods:**

We conducted a cross-sectional descriptive study between 2007 and 2020 with patients clinically and molecularly diagnosed with spinal muscular atrophy who attended a care center. For the functional assessment we used the Hammersmith and Chop-Intend scales and the data were systematized with the Epi-Info, version 7.0 software.

**Results:**

We analyzed 14 patients (42.8 % men). The most prevalent spinal muscular atrophy was type II with 71.4 %. We found phenotypic variability in terms of functionality in some patients with type II spinal muscular atrophy, 37.5 % of whom reached gait. Survival was estimated at 28.6 years.

**Conclusions:**

The findings in the group of patients analyzed revealed that the scores of the revised and expanded Hammersmith scales correlated with the severity of SMA.

La atrofia muscular espinal es una enfermedad neurodegenerativa rara que afecta las neuronas motoras del asta anterior de la médula espinal ^1^^,^^2^. Es el segundo trastorno autosómico recesivo más común, con una incidencia estimada de 1 en 6.000 a 1 en 10.000 nacidos vivos, según estudios en la población general ^3^^,^^4^. En México, la incidencia reportada es de 0,5 a 1 por cada 25,000 nacimientos ^5^. Son pocos los estudios publicados en Colombia sobre esta enfermedad ^2^, por lo que no se cuenta con estadísticas claras sobre su incidencia ni su prevalencia.

La forma clásica de la enfermedad es producto de una variante en los genes que codifican la proteína de la supervivencia de la motoneurona (*SMN1* y *SMN2*), ubicados en el brazo largo del cromosoma 5, y es parte de una duplicación invertida de 500 kb en el cromosoma 5q13. Esta región duplicada contiene, al menos, cuatro genes y elementos repetitivos que la hacen propensa a reordenamientos y deleciones ^6^.

El gen está presente en múltiples copias en el genoma humano, una telomérica, *SMN1*, y varias copias centroméricas, *SMN2*, que se diferencian en un solo nucleótido en el exón 7, el cual se cree que es un potenciador del empalme del exón ^7^; dichas copias son casi idénticas y codifican la misma proteína. Estos genes contienen nueve exones de las copias telomérica y centromérica que se designan como exón 1, 2a, 2b y 3-8. Las mutaciones en la copia telomérica están asociadas con la atrofia muscular espinal, en tanto que las mutaciones en la copia centromérica no producen la enfermedad ^7^.

El gen *SMN2* presenta una tendencia a un empalme génico alternativo (*alternative splicing*) durante la transcripción del ARNm que origina una proteína truncada, con solo el 10 % de la proteína SMN completa. Este gen puede ser un modificador de la enfermedad causada por una mutación en la copia telomérica ^8^. Se cree que los eventos de conversión de genes pueden involucrar los dos genes, lo que lleva a un número variable de copias de cada gen ^7^.

La gravedad de la atrofia muscular espinal clásica es muy variable ^8^, lo que resulta en características clínicas heterogéneas, las cuales pueden clasificarse en cinco fenotipos con base en la edad de inicio y la función motora máxima alcanzada. Debe considerarse como diagnóstico diferencial en la hipotonía congénita ya que, dependiendo de la alteración en el gen, se producen varios fenotipos. El fenotipo 0 se desarrolla en la vida intrauterina y es de suma gravedad ^9^. Le sigue el fenotipo I, correspondiente a la enfermedad más grave en los primeros años. Los fenotipos I y III son identificados en la infancia como intermedios y el fenotipo IV aparece en la adultez ^10^^,^^11^.

La fisiopatología derivada de la historia natural y la función motora de la atrofia muscular espinal, depende de la pérdida temprana de neuronas motoras ^12^. Hasta hace poco, el diagnóstico era tardío ya que, a partir de la sospecha clínica, se requerían una biopsia muscular y un estudio electrofisiológico y había limitaciones en el acceso a la confirmación molecular. Los avances tecnológicos han hecho posible un diagnóstico más temprano y oportuno de la enfermedad, permitiendo la atención oportuna y el manejo interdisciplinario, con la consecuente reducción del estrés del paciente y del cuidador, y un mejor desempeño funcional. Además, el acceso a tratamientos farmacológicos con énfasis en la terapia génica mejora la calidad de vida de los pacientes y el pronóstico de la enfermedad ^13^^-^^17^.

En la evaluación funcional de la atrofia muscular espinal, se emplean diversas escalas (*Chop Intend*, *Rulm*, *Gross Motor Function Measure*), pero la más destacada es la escala de la función motora de Hammersmith (*Hammersmith Functional Motor Scale* - HFMS), cuya eficacia ha sido demostrada en diversos artículos ^18^. La HFMS se utiliza en la población con atrofia muscular espinal sin capacidad de deambulación y, la Hammersmith expandida (HFMSE), en quienes tienen capacidad de deambulación ^19^^-^^21^. En población afectada por el tipo I de la enfermedad, la escala utilizada es la *Chop Intend*.

En este contexto, se hizo un estudio descriptivo para caracterizar clínica y funcionalmente la atrofia muscular espinal en la región centro-occidente del país mediante una encuesta y el uso de escalas de funcionalidad en una serie de casos incluidos en la base de datos de una clínica local.

## Materiales y métodos

Se hizo un estudio descriptivo transversal en el que se caracterizaron variables demográficas y clínicas de 14 pacientes con diagnóstico clínico de atrofia muscular espina,l confirmado mediante criterios clínicos y biopsia muscular sometida a estudio molecular (análisis de la deleción y secuenciación de los genes *SMN1* y *SMN2*). El estudió contó con el consentimiento informado de los pacientes y fue aprobado por el comité de ética de la institución.

La muestra se estableció a partir de la revisión de los registros clínicos, entre el 2007 y el 2020, de pacientes con los siguientes diagnósticos: atrofia muscular espinal, enfermedades de las neuronas motoras, otras atrofias musculares espinales hereditarias, otras atrofias musculares y síndromes afines, neuromiopat**í**a y neuropatía neoplásica.

Una vez confirmado el diagnóstico, cada uno de los pacientes fue evaluado por los investigadores (genetista, fisiatra, fisioterapeuta y médico capacitado) mediante una encuesta, un examen físico exhaustivo y las escalas de la función motora de Hammersmith y la Hammersmith expandida para atrofia muscular espinal de tipos II y III, en tanto que, para la de tipo I, se utilizó la escala *Chop Intend*. Las escalas funcionales fueron utilizadas por un fisiatra y un fisioterapeuta capacitados en su uso.

Los datos se consignaron en un formulario elaborado con todas las variables clínicas y paraclínicas utilizadas para la caracterización clínica, epidemiológica y funcional. La sistematización de los datos se hizo en Epi- Info, versión 7.0, en un formulario validado y diligenciado directamente por uno de los autores. Se exportó una hoja de cálculo al programa Stata™, versión 14.1, y se programó un análisis univariado después de la evaluación mediante un análisis exploratorio. Las medidas numéricas de resumen se expresaron en medianas y percentiles y, las variables categóricas, en frecuencias absolutas y relativas.

## Resultados

Se caracterizaron 14 pacientes que cumplían con los criterios clínicos y contaban con los estudios diagnósticos confirmatorios (prueba molecular para atrofia muscular espinal tipos I, II y III). La prevalencia de la atrofia muscular espinal de tipo I fue de 1/14, la de tipo II fue de 10/14 y la de tipo III fue de 3/14. En el cuadro 1 se muestran las características demográficas de los pacientes analizados, 8 mujeres y 4 hombres; tres fueron adultos empleados. Dos pacientes tenían algún familiar de primer grado con atrofia muscular espinal y ,un paciente, un familiar de segundo grado. No se reportó ningún tipo de consanguinidad en los padres de los pacientes. 

**Cuadro 1:** Características de mográficas de 14 pacientes con atrofia espinal muscular

		Tipo I n=1	Tipo II n=10	Tipo III n=3	Total N=14
Sexo					
	Masculino	0	4	1	5
	Femenino	1	6	2	9
Edad actual					
	Me (P25-P75)	5 (5 - 5)	15 (11 - 27)	15 (14 - 16)	15 (11 - 26)
Ocupación					
	Empleado	0	2	1	3
	Desempleado	0	0	1	1
	Estudiante	0	7	1	8
	Sin datos	1	1	0	2
Inicio de síntomas (meses) Me (P_25_-P_75_)		1 (1 - 1)	9 (8 - 11)	30 (24 - 36)	10 (7,5 - 12)
Edad de diagnóstico (meses) Me (P_25_-P_75_)		11 (11 - 11)	36 (18 - 132)	48 (36 - 348)	36 (18 - 132)
Antropometría	Peso (kg)				
	Me (P_25_-P_75_)				
	Talla (cm)	13 (13 - 13)	36 (17 - 53)	46 (29 - 60)	36 (17 - 53)
	Me (P_25_-P_75_)	105 (105 - 105)	150 (120 - 151)	165 (153 - 165)	150 (120 - 154)
	IMC	11,8 (11,8 - 11,8)	19,5 (12,6 - 21,5)	19,7 (10,7 - 22,1)	19,5 (11,8 - 21,5)
	Me (P_25_-P_75_)				
Diagnóstico molecular					
	*SMN1*	1	9	2	12
	*SMN2*	0	8	1	9
	Sin datos	0	1	0	1
Antecedentes					
	Familiar con AME	0	2	1	3

IMC: índice de masa corporal; AME: atrofia muscular espinal; SD: sin datos;

Me: mediana; P_25_: percentil 25; P_75_: percentil 75;

La mediana de edad al inicio de los síntomas de las atrofias de tipos I, II y III fue de 1, 9 y 30 meses, respectivamente, y en el momento de su diagnóstico, de 11, 36 y 48 meses, respectivamente.

Dos de los 14 pacientes analizados fallecieron antes de la realización de la prueba molecular y su diagnóstico se estableció con base en los síntomas y el reporte de la biopsia muscular. A 12 de los pacientes se les hizo estudio molecular para el *SMN1* y, a 9 para el *SMN2*.

**Figura 1:**
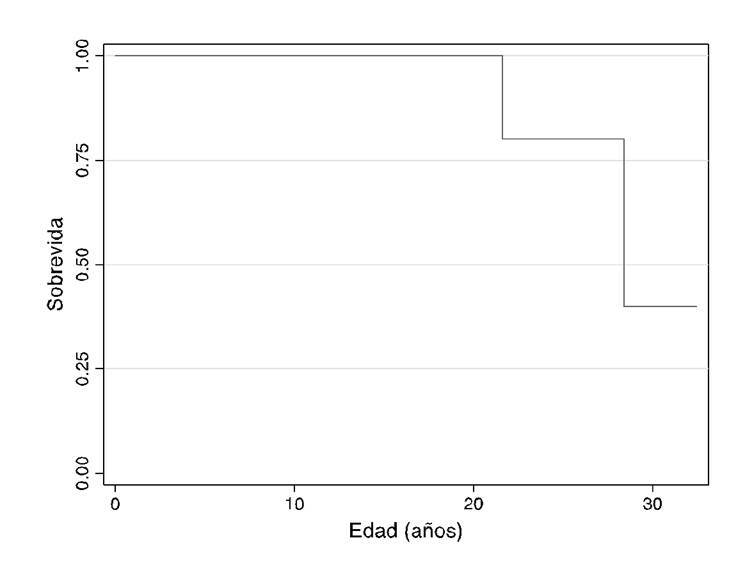
Curva de supervivencia de Kaplan-Meier en pacientes con atrofia muscular espinal

En el cuadro 2 se resumen las manifestaciones clínicas. En el momentode la evaluación, un paciente con atrofia muscular espinal de tipo I presentaba debilidad muscular, hipotonía, disminución de reflejos tendinosos, retraso en el desarrollo, contracturas musculares y disnea. Todos los pacientes con la de tipo II presentaban escoliosis, 9/10 tenían debilidad muscular, hipotonía y disminución de reflejos tendinosos; 8/10 presentaban atrofia muscular, 7/10 retraso en el desarrollo motor, y 5/10, contracturas musculares. Todos aquellos con la de tipo III presentaban hipotonía, debilidad muscular, disminución de reflejos tendinosos, atrofia muscular y displasia de cadera, y 2/3 de este grupo presentaba escoliosis. En la muestra analizada, había un paciente de 5 años con atrofia muscular espinal de tipo I que presentaba gastrostomía y traqueotomía, y requería asistencia respiratoria mecánica permanente. Dos pacientes, uno con la de tipo II y otro con la de tipo III, fallecieron por causas relacionadas con la enfermedad. Se analizó la curva de supervivencia y se estimó una supervivencia de 28,6 años (IC95% 24,8-32,5) (figura 1).

**Cuadro 2:** Características clínicas de los pacientes con atrofia espinal muscular en el momento de la evaluación

	Tipo I n	Tipo II n	Tipo III n	Total n
Control de esfínteres	0	7	2	9
Debilidad muscular	1	9	3	13
Hipotonía	1	9	3	13
Disminución de reflejos tendinosos	1	9	3	13
Atrofia muscular	1	8	3	12
Fasciculaciones linguales	0	6	2	8
Retraso del desarrollo motor	1	7	3	11
Contracturas musculares	1	5	1	7
Disnea	1	5	1	7
Malnutrición	0	1	0	1
Displasia de cadera	0	6	1	7
Escoliosis	1	10	2	13
Apnea del sueño	0	3	0	3
Gastrostomía	1	0	0	1
Traqueostomía	1	1	1	3
Asistencia respiratoria mecánica	1	1	0	2
Oxigenoterapia	0	1	0	1
Antecedente quirúrgico ortopédico	0	4	2	6
Adquisición de marcha	0	3	3	6
Edad de pérdida (años) de marcha				
Me (P_25_-P_75_)	SD	11 (10 - 13)	3 (3 -11)	10,5 (3 - 11)

Me: mediana; RIQ: rango intercuartílico; SD: sin datos

En la evaluación funcional de los pacientes con atrofia muscular espinal de tipo I, se empleó la escala *Chop Intend* y, en la de los tipos II y III, se utilizaron las versiones revisada y expandida de la escala Hammersmith, cuya puntuación máxima es 40 en la primera y 66 en la segunda. En la evaluación funcional de los pacientes con atrofia de tipo II, la mediana del puntaje fue de 12,3 (rango intercuartílico: 19,5) y, en aquellos con la de tipo III, esta fue de 0,0 (rango intercuartílico: 26), sin diferencias estadísticamente significativas (p=0,536).

**Figura 2:**
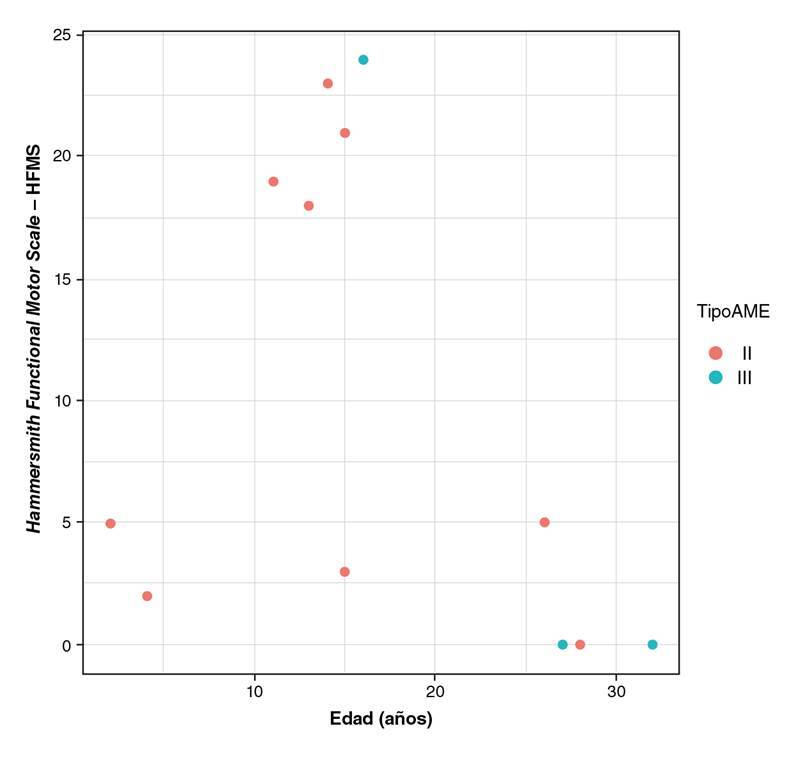
Relación funcional de pacientes con atrofia muscular espinal de acuerdo con la edad

El puntaje funcional en el paciente de la cohorte con atrofia muscular espinal de tipo I, fue de 3 a la edad del análisis (5 años). En 5 de los 9 casos de la de tipo II, se obtuvieron puntajes en la escala de Hammersmith que oscilaron entre 0 y 5; 4 de ellos obtuvieron valores mayores, cercanos a los puntajes funcionales de aquellos con la de tipo III (entre 18 y 20). Dos de los pacientes con atrofia de tipo III obtuvieron un puntaje de 0. Se encontró una correlación entre la edad en el momento de la evaluación y el puntaje funcional en la escala de Hammersmith (figura 2).

## Discusión

La atrofia muscular espinal es una condición rara cuyo manejo diagnóstico ha tenido cambios sustanciales en los últimos años con el advenimiento de las nuevas técnicas moleculares que han relegado la biopsia muscular a ser un estudio de segunda línea ^19^. Dos de los pacientes estudiados habían sido diagnosticados años atrás mediante biopsia muscular y fallecieron antes de poder practicarles la prueba molecular, lo que hubiera permitido un adecuado asesoramiento genético familiar.

Los estudios demográficos de la atrofia muscular espinal a nivel mundial se han realizado en pequeñas cohortes con características heterogéneas, en su mayoría en países europeos. Esta situación ha llevado a que el porcentaje reportado para los subtipos sea diferente en cada estudio. Por ejemplo, en el de Verhaart, *et al*., en el que se unificaron los datos de prevalencia e incidencia de la enfermedad y sus diferentes subtipos disponibles a nivel mundial, los autores concluyeron que la prevalencia general era de 1 a 2 por cada 100.000 nacidos vivos, siendo las atrofias musculares de tipo II y III las más frecuentes, con el 75 % de casos, en tanto que la de tipo I registró el 28% de los casos, aproximadamente ^4^. En una descripción de 29 casos en Medellín, se encontró que la forma más común era la atrofia muscular espinal de tipo II, con el 62,1 % de los casos ^2^.

En el grupo de pacientes reportados en este estudio se encontró que la forma más común fue la atrofia muscular espinal de tipo II (10/14), seguida de las de tipos III (3/14) y la tipo I (1/14), lo que coincide con los hallazgos publicados a nivel mundial. Este bajo porcentaje de prevalencia de la atrofia muscular espinal de tipo I se explica, como en otros estudios, por las bajas tasas de supervivencia de los pacientes con este fenotipo.

La atrofia muscular espinal se clasifica en diferentes fenotipos, pero se mantienen, en general, los grupos de atrofia muscular espinal de los tipos I, II, III y IV, aunque en algunos se hace una descripción más detallada y se introducen subdivisiones. Por ejemplo, en el 2005, Bertini, et al., subdividieron la atrofia muscular espinal de tipo I en tres grupos: 1A o cero, IB y IC, según las manifestaciones clínicas y la gravedad en el momento del nacimiento ^20^.

La de tipo 1A es la más grave, ya que el neonato presenta contracturas articulares y escasos movimientos, y hay necesidad de darle soporte respiratorio. El tipo 1B se caracteriza por un control deficiente de la cabeza y dificultad para manejar las secreciones orales, y su pronóstico es intermedio. En el tipo 1C hay control de la cabeza y posibilidad de sentarse con apoyo, por lo que el pronóstico es mejor ^20^.

En este estudio solo hubo un paciente de 5 años con atrofia muscular espinal de tipo IC, cuyos síntomas habían aparecido antes del año; presentaba debilidad muscular grave, hipotonía, arreflexia y atrofia muscular, y requería asistencia respiratoria mecánica permanente y alimentación por gastrostomía.

En las cohortes de pacientes con atrofia muscular espinal del tipo I descritas en la literatura especializada, la edad mediana de muerte ha sido de 13,5 meses y todos los mayores de 12 meses han requerido soporte respiratorio y alimentación por gastrostomía ^21^. Las características de la de tipo I de este estudio no son comparables con las reportadas en otros estudios, pues se trató de un solo paciente con una supervivencia por encima de la usualmente reportada, lo que respondería a la disponibilidad de nuevas terapias. El caso, además, podría clasificarse en el subgrupo 1C del tipo I ^22^.

En la cohorte constituida por 240 pacientes con atrofia muscular espinal de tipo II descrita por Zerres, *et al*. ^23^, ninguno alcanzó la marcha independiente y la mayoría desarrollaron escoliosis y contracturas tempranamente. En la cohorte analizada en este estudio, todos los pacientes con atrofia muscular espinal de tipo II desarrollaron escoliosis, 5/10 presentaban contracturas musculares y 3/10 alcanzaron la marcha. Según las escalas de Hammersmith revisada y expandida, entre estos casos había variedad fenotípica y se obtuvieron puntuaciones bajas y medias en aquellos con la atrofia de los tipos II y III, resultados que demuestran la capacidad de dichas escalas para discriminar la capacidad motora y funcional entre los diferentes fenotipos de atrofia muscular espinal en los pacientes de esta región del país.

Todos los pacientes con atrofia muscular espinal de tipo III descritos en la cohorte de Zerres, *et al.*^23^, lograron caminar sin ayuda y el curso de la enfermedad se caracterizó por una progresión lenta y con períodos de aparente detención del deterioro. De estos 320 pacientes, únicamente el 10% falleció y las muertes no estuvieron relacionadas con la enfermedad. En este grupo, las características clínicas más importantes fueron el desarrollo de escoliosis en el 66,7 % de los casos y de displasia de cadera en el 33,3 % y, como ya se dijo, todos los pacientes lograron la marcha y el 66,6 % de ellos estudiaba carreras técnicas o profesionales en el momento de la evaluación ^23^. En el presente estudio, uno de los pacientes con atrofia muscular espinal de tipo III falleció y su muerte estuvo relacionada con la enfermedad.

Debido a que los síntomas de la atrofia muscular espinal de tipo III aparecen mucho más tardíamente, esta suele diagnosticarse más tarde que las de los tipos I y II. Lin, *et al.*^13^, en su revisión sistemática de estudios en Asia, Europa y Norteamérica, informaron que el tiempo transcurrido entre la aparición de los síntomas y el diagnóstico de la enfermedad fue de 3,6, 14,3 y 43,6 meses para los tipos I, II y III, respectivamente. En México, Urrutia, *et al.*, informaron que el retraso en el diagnóstico había sido de 6, 38 y 42 meses para los tipos I, II y III, respectivamente, datos similares a los del presente estudio, con 11, 36 y 48 meses para los tipos I, II y III, respectivamente ^5^.

Debe mencionarse que los pacientes con el tipo III llegan a la adolescencia y la adultez logrando estar de pie o caminar sin apoyo, auqnue la gran mayoría de ellos pierde la deambulación con el tiempo ^24^. Esto se correlaciona con los datos encontrados en nuestra serie de casos, en la que se evidenció una mayor proporción de pacientes adolescentes y adultos con atrofia muscular espinal de los tipos II y III.

La mediana de supervivencia estimada (50%) para los pacientes con atrofia muscular espinal analizados en el presente estudio, fue de 28,6 años, dato similar a los de otros estudios con supervivencia de 68,5 % a los 25 años ^23^.

El antecedente de familiares con atrofia muscular espinal y la consanguinidad, constituyen una alerta para el médico de atención primaria, por lo que esto debe averiguarse siempre en los casos de niños hipotónicos ^5^. En un estudio realizado en México, el porcentaje global de consanguinidad fue de 10,4 % y en comunidades urbanas, de 9 % ^5^; además, dos de las 31 familias reportaron consanguinidad. En un estudio en Medellín, se evidenció que el 13,7 % de los pacientes tenía familiares afectados ^2^.

En el presente estudio, se encontró que 3/14 de los pacientes tenía familiares afectados. Llama la atención que toda la región comparte la ancestralidad de padre español y madre indígena, así como una historia de pocas familias con numerosos hijos que poblaron toda la región, por lo que podría existir un efecto fundador con múltiples portadores que solo podría detectarse mediante un tamizaje neonatal para confirmar su presencia y el riesgo de la enfermedad en la zona ^25^. Sin duda, ello contribuiría al diagnóstico temprano y a un tratamiento precoz, incluso desde la etapa presintomática, con incidencia en la evolución de la enfermedad y mejoras significativas en la funcionalidad y la calidad de vida de los pacientes.

En términos de funcionalidad, en las cohortes de pacientes con atrofia muscular espinal de tipo I, se describen puntajes promedio en la escala *Chop Intend* de 5,3 (± 2,8) a los 24 meses de edad ^26^ y, en el presente estudio, el niño con atrofia muscular espinal de tipo I obtuvo una puntuación de 3 a los 5 años. En la evaluación funcional de los pacientes con los tipos II y III con las versiones revisada y expandida de la escala Hammersmith, no se encontraron diferencias importantes en las puntuaciones promedio en los de tipo II comparados con los de tipo III. Según lo reportado sobre las propiedades de la escala, esta puede discriminar los diferentes tipos de presentación clínica de la enfermedad ^27^; no obstante, esto podría no haberse evidenciado debido al reducido tamaño de la muestra, ya que solo tres pacientes tenían atrofia muscular espinal de tipo III. En el presente estudio, además de describir la funcionalidad de los pacientes, no se encontró correlación de las puntuaciones obtenidas en la escala con el número de copias de *SMN2* presentes.

En la revisión de los estudios publicados en Colombia, no se encontró ninguno que describiera la funcionalidad en pacientes con atrofia de los tipos II y III, según la escala de Hammersmith y la de Hammersmith expandida. En la mayoría de los estudios en que se emplearon escalas funcionales para estos tipos de atrofia muscular, se analizan los cambios en los puntajes funcionales en un período variable de acuerdo con la cohorte. En general, en las cohortes reportadas en la literatura, la reducción en los puntajes funcionales empieza a ser evidente después de los 12 meses de seguimiento en casos de atrofia de los tipos II y III no tratados con nusinersen ^28^.

La variabilidad fenotípica de la funcionalidad en 4 de los 9 pacientes con atrofia muscular espinal de tipo II, encontrada en el presente estudio con puntajes cercanos a 20 podria deberse a la variabilidad en el numero de copias *SMN2* en pacientes heterocigotos es similar a la reportada por otros autores, esto, como la ^29^. La identificacion de variantes con papel modificador como la c. 460C> T en el gen *SMN2* relacionada con un fenotipo de leve gravedad, y las variantes c.770_780dup y c.734_735insC aportan a un fenotipo variable.

En otros estudios se menciona el papel importante de algunos genes cercanos a *SMN1* y *SMN2* que, al parecer, son claves en la modificación de la gravedad de la enfermedad, como es el caso de los genes *NAIP*, *H4F5*, *GTF2H2* y *PLS3*^31^^-^^33^. Estos hallazgos permiten determinar que, en el grupo de pacientes colombianos analizados en la presente serie, los puntajes de la escala de Hammersmith revisada y la expandida se correlacionaron con la gravedad de la enfermedad.

Actualmente, las pruebas moleculares permiten el diagnóstico temprano y no invasivo de la atrofia muscular espinal, que antes se hacía con base en criterios clínicos y familiares y la biopsia, como lo evidencian los pacientes de mayor edad en el presente estudio, quienes fueron diagnosticados de forma tardía y no contaban con los datos sobre el número de copias de *SMN2*; además, un paciente con diagnóstico clínico, familiar y biopsia falleció antes de que se le practicara la prueba molecular.

En el presente estudio, hubo una gran proporción de pacientes adultos con atrofia muscular espinal, profesionales que tienen una vida activa y socialmente productiva, por lo que se beneficiarían de los tratamientos disponibles actualmente, logrando 1 o 2 puntos en la escala Hammersmith expandida, lo que significaría una mejoría clínicamente significativa dado su riesgo de deterioro progresivo y muerte. La asociación entre el diagnóstico molecular y el manejo interdisciplinario temprano de los pacientes, sumada al análisis funcional mediante pruebas disponibles, permite el correcto manejo de los pacientes con atrofia muscular espinal.

En estudios posteriores se debe evaluar objetivamente la prevalencia de esta enfermedad en el eje cafetero. Hoy no se cuenta con tamizaje neonatal de la atrofia muscular espinal en la región, aunque la cantidad de pacientes amerita hacerlo dado el alto grado de consanguinidad en la población. En los pacientes analizados en este estudio, se requieren futuros análisis genéticos que pueden detectar variantes modificadoras del fenotipo.

El diseño transversal del presente estudio constituye una limitación, ya que los pacientes se analizaron en una única oportunidad, lo que no permitió evaluar los cambios funcionales a través del tiempo.
